# Attrition and success rates of accelerated students in nursing courses: a systematic review

**DOI:** 10.1186/s12912-016-0145-7

**Published:** 2016-04-08

**Authors:** Sheila Anne Doggrell, Sally Schaffer

**Affiliations:** School of Biomedical Sciences, Faculty of Health, Queensland University of Technology, GPO 2343, Brisbane, QLD 4001 Australia

**Keywords:** Accelerated nursing students, Accelerated programs, International students, Non-university graduate students, University graduates

## Abstract

**Background:**

There is a comprehensive literature on the academic outcomes (attrition and success) of students in traditional/baccalaureate nursing programs, but much less is known about the academic outcomes of students in accelerated nursing programs. The aim of this systematic review is to report on the attrition and success rates (either internal examination or NCLEX-RN) of accelerated students, compared to traditional students.

**Methods:**

For the systematic review, the databases (Pubmed, Cinahl and PsychINFO) and Google Scholar were searched using the search terms ‘accelerated’ or ‘accreditation for prior learning’, ‘fast-track’ or ‘top up’ and ‘nursing’ with ‘attrition’ or ‘retention’ or ‘withdrawal’ or ‘success’ from 1994 to January 2016. All relevant articles were included, regardless of quality.

**Results:**

The findings of 19 studies of attrition rates and/or success rates for accelerated students are reported. For international accelerated students, there were only three studies, which are heterogeneous, and have major limitations. One of three studies has lower attrition rates, and one has shown higher success rates, than traditional students. In contrast, another study has shown high attrition and low success for international accelerated students. For graduate accelerated students, most of the studies are high quality, and showed that they have rates similar or better than traditional students. Thus, five of six studies have shown similar or lower attrition rates. Four of these studies with graduate accelerated students and an additional seven studies of success rates only, have shown similar or better success rates, than traditional students. There are only three studies of non-university graduate accelerated students, and these had weaknesses, but were consistent in reporting higher attrition rates than traditional students.

**Conclusions:**

The paucity and weakness of information available makes it unclear as to the attrition and/or success of international accelerated students in nursing programs. The good information available suggests that accelerated programs may be working reasonably well for the graduate students. However, the limited information available for non-university graduate students is weak, but consistent, in suggesting they may struggle in accelerated courses. Further studies are needed to determine the attrition and success rates of accelerated students, particularly for international and non-university graduate students.

## Background

Ongoing nursing shortages in many countries are driving the need to train more nurses [1–3]. In addition to increasing demand, the nursing shortages are partly due to loss of nurses from the profession [4, 5]. Also, in the United States of America (USA), it is known that there are major differences in access and care outcomes for ethnic and racial minorities, and there is evidence to suggest that a diverse healthcare workforce will improve health disparities of ethnic and other socially disadvantaged groups [6]. Thus, in many countries, universities face the challenge of producing increasing or similar numbers of students and diversifying the population of nurses.

One of the universal primary strategies for increasing participation in nursing education, including in North America and Australia, has been the introduction of accelerated or second degree nursing programs. Accelerated nursing programs are usually shorter than the three year traditional/baccalaureate nursing programs, and can be divided into two types. Firstly, there are specific accelerated programs with a curriculum designed for accelerated students only, receiving mainly graduates from an area other than nursing. Accelerated baccalaureate nursing programs are an example of specific programs for accelerated students, and these are common in the USA [7] and Canada [8]. Secondly, there are accelerated programs where the students join the traditional students for part of the traditional/baccalaureate program, and this is the model commonly used in Australia, where the accelerated students undertake the last two years of the traditional program. A similar model was introduced in the United Kingdom (UK) in September, 2013 [9].

Students entering accelerated programs (accelerated students) are granted academic credit for prior learning or experience. These accelerated nursing programs recruit a population of students that are often different from the traditional nursing students, who are predominantly students who have completed high (secondary) school. Thus, the students entering accelerated programs are often older, and receive academic credit for prior learning in a related or unrelated field, or recognition of an equivalent learning in the form of prior work place or life experience [2, 7].

There is a comprehensive literature about the academic outcomes (e.g., attrition and success) of students in traditional/baccalaureate nursing programs [10, 11], but much less is known about the students in accelerated nursing programs. We have evidence that the accelerated students in the program at our university have higher attrition rates than the traditional students [12, 13]. Thus, we decided to undertake a systematic review to determine whether this was a common finding.

We divided the accelerated students into three groups, based on how they have been identified in the previous literature. Firstly, there are the international students. The international students have some form of higher education, are studying in a foreign country, and some are English-as-a-second language (ESL) students. The second group of accelerated students are students, who have previously graduated from a university in a non-nursing course with a degree, and are studying in their native country. The third group of accelerated students are the non-university graduates, who are studying in their native country, but have not graduated with a degree from a university, but have either studied nursing, but not to degree level (e.g., diploma, associate degree), or have work experience which is considered equivalent to study to a non-degree level. Pathways for non-university nursing graduates to become registered nurses are available in several countries including Australia [14], New Zealand [15], USA and Canada [16] and the UK [17].

The objective of this review is to report the outcomes of attrition and success rates for the populations of international, graduate and non-university graduate accelerated students in Bachelor of Nursing courses, in comparison with traditional students. In previous literature and this review, attrition rates are the rates of students withdrawing from the course/program and leaving the university. The success rates of students have been reported in different ways in the literature, and consequently in this review we report whether the success was internal (e.g., at the end of the unit/course/program) or external (e.g., National Council Licensure Examination for Registered Nurses, NCLEX-RN). In some studies the attrition and success rates of accelerated nursing students are compared to the traditional students.

## Methods

**Fig. 1:**
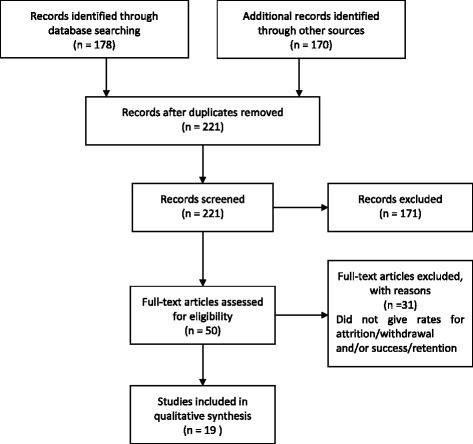
PRISMA flow diagram of the literature search for the review

For this systematic review of attrition and success rates, Google Scholar, Pubmed, and Cinahl were searched using the search terms ‘accelerated’ or ‘accreditation for prior learning’ or ‘fast track’ or ‘top up’ and ‘nursing’. These search terms led to papers on graduate, international and non-graduate students, and were combined with ‘attrition’ or ‘retention’ or ‘withdrawal’ or ‘success’. The PRISMA flow diagram of the literature search for the review is given in Fig. 1. The search was conducted from 1994 to January, 2016, and after completion, duplicate entries were removed. English only abstracts were read. The two authors independently screened the titles, abstracts and/or articles. A large number of records were excluded from the abstracts (171), and further 50 were excluded from full-text, as although they did mention attrition/withdrawal/retention or success, they did not provide data on the subject of the review, which was the rate of these. Thus, most of the studies initially screened were of student experiences, predictors and perceptions of success, learning strategies or masters programs. We also excluded articles that did not identify accelerated students as international, graduate and/or non-university graduate accelerated students, and studies that did not separate international graduate accelerated from international non-graduate undergraduate students.

Data was collected relating to the country of the study, study methodology, numbers of traditional and accelerated students, and whether they were international, graduate and/or non-university graduate students. Rates of attrition/withdrawal/retention, and success, measured internally or at NCLEX-RN, were collected. Limitations, including any bias, were identified for individual studies.

One abstract did not separate the rates of success for the different groups of accelerated students, was not available as full-text, and this record was excluded from our systematic review. Fifty full-text articles were available and assessed for eligibility. Of these studies, 31 did not give rates of attrition/withdrawal and/or rates of success/retention. Nineteen articles that included rates of attrition/withdrawal/retention or success were identified. As these were of variable quality, it was decided to include all of them with discussions of their strengths and weaknesses. As the majority of the studies with the graduate students provided similar results, they were combined. However, there was considerable heterogeneity in the studies of international students, and only three studies with the non-university graduate accelerated students, and we decided not to combine the studies, but to provide details of the studies.

## Results

### International students

Accelerated international nursing students have some form of higher education and are undertaking a Nursing course in a foreign country. Some, but not all international students have ESL.

The literature relating to international accelerated nursing students often does not distinguish between students in traditional and accelerated programs. Consequently, we only found three studies from the literature search that clearly identified that they related to international students in accelerated programs, and one of these studies suggested that international students had low attrition, one study showed high success, whereas the other study showed high attrition and low success (Table 1).

**Table 1:** Attrition and/or success rates of international students entering BNursing course as accelerated students

Country	Subjects	Attrition and/or success rates	Reference
US	37 Saudi Arabian citizens with proficiency in English	8 % attrition and 92 % success	[18]
Australia	52 of 61 students who spoke a language other than English at home	Higher GPA at the end of first semester than traditional students	[19]
Australia	20 students from Asian countries	20 % attrition and 70 % success	[20]

The study showing a low attrition rate was completed in 2007. The international students were 37 Saudi Arabian citizens, mainly male graduates in science or a related field, enrolled in an accelerated nursing program in the USA over a ten year period [18]. Students had to show proficiency in English to undertake the course [18]. In this study, there was a 92 % success rate in the Bachelor of Science in nursing program, and an eight percent attrition rate [18]. The weaknesses of this study [18] are that it does not have a comparison group, and that it is limited to a small specific population, which makes it difficult to extrapolate the results to international students in general.

The study showing high success rates for accelerated international students was performed in Australia [19]. This study had a sample of 61, and 52 students were international students who spoke a language other than English at home, and scored at the lower end on a language acculturation scale [19]. However, these students performed better academically (Grade Point Average, GPA) at the end of first semester, than nursing students undertaking the traditional course at the same university. The authors acknowledged that a weakness of their study was that the international/accelerated and traditional courses were not equivalent, with the traditional course having more written assignments in the first year and this may have biased the results in favour of the international students [19].

The study showing high attrition rates/low success rates, evaluated an accelerated program attended by 20 international nursing students (all from Asian countries) in an Australian university [20]. This program was devised to address some of the problems facing students studying in a foreign country, namely English language proficiency, cultural and communication difference and unfamiliarity of the health care system [20]. However, only 14 students progressed to second semester, which is success rate of 70 % in first semester. Four of the students who failed did not re-enrol, giving an attrition rate of 20 % [20]. The major weaknesses of this study [20] were that there was a low sample size and that there was no comparison to outcomes for traditional students.

### Graduate students

Graduate students given accelerated entry into nursing courses, are students who have previously graduated from university in a non-nursing course, and are studying in their native country. All of the studies of the attrition rates of graduate students have been undertaken in the USA (Table 2), and the majority of studies of success of graduates (8 of 11) have been performed in the USA (Tables 2 and 3). The majority of these studies have shown that the attrition and success of graduate students is better or similar to that of traditional students.

**Table 2:** Attrition rates (and success rates, if given) of graduates entering BNursing courses as accelerated students

Country	Subjects	Attrition and success rates	Reference/s
US	Compared attrition and success rates from 13 month accelerated second-degree nursing program (226 students) with separate traditional program over five year period (204 students).	Attrition rates, 3 % for graduates, 6-7 % attrition rate for traditional students. The NCLEX pass rates were higher for accelerated than the traditional students.	[21]
US	Measured attrition rates and performance of 363 graduates in accelerated program over 5 years	Attrition rates of about 14 % for second degree students and of the remaining students 88 % passed NCLEX on first attempt. Compared with 22 % attrition rate for traditional students.	[22]
US	Between 157 and 168 applicants were interviewed per year for accelerated entry over four years, and between 11 and 14 were denied entry	Attrition rates of students in the accelerated program was 10–15 %; averaged 20 to 30 % for traditional students	[23]
US	Initially compared graduated (71) in accelerated second-degree nursing program with traditional program (76 students) over six year period. Subsequent study of further 81 graduates.	Attrition rates of about 10 % from both courses. Passing rates for the NCLEX-RN were 84 and 85 %.	[24, 25]
US	Compared attrition and success rates from 13 month accelerated second-degree nursing program (52 graduates) with a traditional program (172 students).	Attrition rates, 12 % for graduates, not given for traditional students. Passing rates were similar in the courses (~90 %) and in NCLEX.	[26]
US	39 graduate students over 2 years	Graduation rates (combined attrition and success) rates of 29 and 50 % in 1st and 2nd years, respectively. Commented that this was much higher than for the traditional program.	[27]

**Table 3:** Success rates of graduates entering BNursing courses as accelerated students (data not given in Table 2)

Country	Subjects	Success	
Australia	130 traditional students and 34 accelerated students	Internal grades were not significantly different between these groups	[28]
Canada	87 traditional and 16 accelerated students	Overall internal grades were not significantly different	[29]
US	46 traditional students and 48 graduate accelerated students	Accelerated students had higher marks internally. None of the traditional students failed the NCLEX-RN, but two of the accelerated students did	[30]
US	32 traditional students and 29 graduate accelerated students	In internal examinations, the accelerated students performed significantly better than the traditional students	[31]
Australia	471 traditional students and 259 graduate students including 84 international students	The graduate accelerated students obtained higher internal marks than the traditional students	[32]
US	33 traditional students and 40 graduate accelerated students	Five of the traditional group failed the NCLEX the first time, compared to 3 from the accelerated student group, and this was not significantly different	[33]
US	29 traditional and 27 graduate accelerated students	Accelerated students had a 90 % passing rate in the NCLEX-RN, compared to 70 % of the traditional students	[34]

For attrition rates, six separate studies describing attrition rates of graduate students from accelerated programs were identified (Table 2; 21–27). Four of these studies had the strength of comparing the attrition rates of graduate students from accelerated programs with those of students completing a traditional nursing course, with three studies suggesting the attrition rates were lower (less than 15 %) [21–23] and one study suggesting they were the same as the traditional nursing course (10–12 %) [24, 25].

One of two other studies gave the attrition rate as being 12 % for graduates, but had the weakness of not giving attrition rates for traditional students [26], and thus it is not possible to say whether this attrition rate was with respect to traditional students. The other study was of 39 students did not report on attrition, but reported on graduation on time, which was the combination attrition and success, and stated that 29 to 50 % did not graduate on time. The authors commented that this was a much lower rate than from the traditional program. However, this study also reported that a high percentage of these students were continuing in the program [27]. As these students may have graduated eventually, graduation time is not a measure of attrition. The other weaknesses of this study [27] were a small sample size, and there was no direct comparison with traditional students.

Four of the six studies reporting low attrition rates for graduate students also reported NCLEX-RN success rate, and had the strength of comparing these rates to those of traditional students [Table 2]. Three of these reported high success rates of accelerated graduate students in the NCLEX-RN ([21, 22, 24]; Table 2), and one reported similar success in the NCLEX-RN for accelerated and traditional students ([26]; Table 2).

In addition, seven other studies describing the success (but not the attrition) of accelerated graduate students were identified, and the success data is given in Table 3 [28–34]. These seven studies have used either internal grading or the NCLEX-RN as a measure of success, and have the strength of comparing the success of accelerated graduate to traditional students.

Combining the studies measuring success from internal grading in Table 2 [21, 22, 24–26] and Table 3 [28, 29, 31, 32], we show that accelerated graduate students do as well as traditional students in Australia [28] and Canada [29] or better than traditional students in the USA [28, 29, 31, 32]. Studies reporting pass rates at NCLEX-RN in the USA also report the graduates do similarly [24, 26, 30, 33] or better [21, 22, 34] than the traditional students.

### Non-university graduate students

Non-university graduate students are students, who have not graduated from a university with a degree, but have either studied nursing to a diploma or associate-degree level, but not to degree level. Alternatively, non-university graduate students can be students who have work experience in a nursing area which is considered equivalent to study to a non-degree level. These non-university graduate accelerated students are studying in their native country.

A search of the literature revealed only three studies reporting the attrition rates for diploma students accelerated into a degree nursing program. Two of these were Australian studies and one of these was a Canadian study, and all report higher attrition rates for accelerated students. One of these studies, completed in Western Australia, was weakened by the fact that the 112 accelerated students were rural and studying externally, but they were compared to the internal traditional students [35]. To be enrolled, the accelerated students had to have one years’ experience as an enrolled nurse, and 91 % had college or hospital-based qualifications [35]. The students were required to attend a two week orientation program and then a two year course [35]. The attrition rates, from the accelerated external and traditional internal courses were compared, and were 26 % from the accelerated student course compared to a 17 % from the traditional program [35]. The authors noted that the higher attrition rate for the external cohort was consistent with the literature relating to external courses and online learning [35]. They also suggested that a difference in the mentor/s for the courses may have contributed to bias in attrition rate, as the internal traditional students were more satisfied with their mentor/s than the external accelerated students [35]. Because of the different modes of delivery in this study and different mentors, it is difficult to determine whether the differences in academic background contributed to the greater attrition of the non-graduates.

At a university in Queensland, diploma students were accelerated into the second year of a degree program at the northern small campus, by joining in with the second year of a traditional program, with no preparation [12]. A third of the 33 diploma students, who were accelerated into the second year of a traditional nursing course, withdrew from the science units and left the university [12]. In contrast, the attrition rate for the 28 traditional students in the same course was less than 3 % [12]. The strength of this study [12] was that it is a direct comparison of diploma and traditional students, but a weakness was that it has a low sample size.

The most recent study of accelerated diploma students was from Canada, and was of 432 diploma students, who undertook a bridging program, which they were given credit for when entering the Bachelor of Science in Nursing program [36]. In this cohort, 85 students formally withdrew (~20 %), and a further 45 discontinued enrolment (~10 %) [36]. The major weakness of this study [36] was that there is no direct comparison to the traditional students.

## Discussion

### International students

There are major limitations to the three studies with international students, which may contribute to the variable and inconclusive results for success and retention rates in this population. Thus, the study of Saudi Arabian citizens [18] showing high success and low attrition did not have a comparison to traditional students, and we cannot be certain that the attrition/success rates were better than the traditional students. Consequently, studies with comparisons to traditional students are necessary to confirm, or otherwise, these findings to date of the attrition and success rates of accelerated Saudi Arabian students. The other two studies of international accelerated students were undertaken in Australia, and do have a comparison to traditional students, which gives them a higher level of proof. However, in one of the studies, the comparison to the traditional students is not a direct comparison, and this is a limitation. Given the limitations of these studies, it is perhaps not surprising that there are mixed results with one showing low success rates for the international students [20] and the other showing better success than the traditional students [19].

Another major limitation of all three studies [18–20] is that they have small cohorts of international students: 37, 52 and 20, respectively. This suggests that further and larger studies need to be undertaken to determine the attrition and success of international students as accelerated students in nursing degrees. Furthermore, as international students study as accelerated students in other countries (e.g., Canada, UK), which may have different curricula to the USA or Australian courses, the attrition and success of the international students in these countries need to be evaluated.

### Graduate students

There is strong evidence that graduate students entering Bachelor of Nursing (BNursing) courses as accelerated students have similar or better outcomes in attrition and success rates as traditional students. At the present time, graduates entering accelerated programs in USA universities perform as well as or better than traditional students. In the USA, science is a prerequisite for accelerated graduate nursing programs with bridging/pre-requisite courses being offered for students who do not hold the necessary entry science requirements [7]. Thus, it seems likely that the present accelerated graduate students in the US studies may have the necessary science requirements to succeed in accelerated programs. Indeed, four studies with low attrition (less than 14 %) stated that graduates were required to have a background in anatomy, physiology and microbiology [21, 22, 24–26].

In Australia, there is not a national requirement, for the graduate students to have a background in science to be accelerated. The two small studies, which suggested that graduate students do as well as the traditional students, did not disclose whether the students had a background in science [28, 30]. Further larger studies are needed to determine whether graduate students have similar attrition and success to traditional students in Australia, and it would also be of interest to know whether having a science background was a factor in this. At present, we do not know whether graduate students are as successful as accelerated nursing students in countries, other than the USA and Australia, and this needs to be evaluated.

A recent integrative literature review considered outcome measures, including academic outcomes, for graduates accelerated into nursing, only included 3 academic outcomes studies; 26, 31, 33, which were all performed in the USA [36]. The present systematic review includes these 3 studies, and includes 5 further academic outcome (success) studies in the USA; 21, 22, 24/25, 28, 30, 34, and two from Australia [28, 32] and one from Canada [29]. The difference is mainly due to the integrative review only including studies from the last six years [37], whereas this systematic review was from 1994, and five of the studies were published between 1994 and 2008; 24/25, 28, 29, 30, 34. There are three recent studies in our systematic review that were not included in the integrative review; 21, 22, 32.

The integrative review showed that in the academic outcome studies they considered (26, 31, 33), the graduate students, who had been accelerated, had similar or better success rates to the traditional students [37], and we have confirmed this. The integrative review suggested that the findings from these studies should be treated with caution, as there were methodological limitations to the three studies [37]. With the addition of eight further studies in our systematic review, it shows that despite methodological limitations in some studies, it is a consistent finding that graduates accelerated into nursing have similar or better success rates than traditional students.

### Non-university graduate students

Although the studies of non-university graduate students are few, and they have weaknesses, they are consistent in finding poor outcomes in BNursing courses for this population. Thus, only two Australian studies and one Canadian study have reported on attrition rates for diploma students, they have all indicated that they are higher than for traditional students [12, 35, 36]. A weakness is that only one of these studies had a direct comparison to traditional students, but the same study was limited by a small cohort [12]. Collectively these studies show that interventions may be required to improve the attrition and success of diploma students accelerated into nursing degrees. As attrition and success have not been reported for diploma or associate degree students or their equivalents in countries other than Australia and Canada, it is not known how these students fare as accelerated students, and this needs to be assessed.

## Conclusions

The paucity of information available makes it unclear as to the attrition and/or success of international accelerated students in accelerated programs. The information we have for graduate accelerated students, suggests that accelerated programs are working reasonably well for the university graduates. Only a few studies have been undertaken with non-university graduate students, and these studies suggest that these students have high attrition and low success in accelerated nursing programs.

### Availability of data and materials section

Data supporting our findings can be found by using our literature search methods or directly from us by application.

## Abbreviations

BNursing: Bachelor of Nursing
ESL: english-as-a-second language
GPA: grade point average
NCLEX-RN: National Council Licensure Examination for Registered Nurses
UK: United Kingdom
USA: United States of America
